# Supplementary material for: Luminescence Properties of an Orthorhombic KLaF4 Phosphor Doped with Pr3+ Ions under Vacuum Ultraviolet and Visible Excitation
Source: Materials (Basel). 2024 Mar 19;17(6):1410. doi: 10.3390/ma17061410 (PMC10971862; doi:10.3390/ma17061410)
Supplement: Supplementary file 1 [file materials-17-01410-s001.zip › materials-2903733-supplementary.pdf]

Supplementary Materials

Luminescence Properties of an Orthorhombic $KLaF_4$ Phosphor Doped with Pr^{3+} Ions under Vacuum Ultraviolet and Visible Excitation

Patrycja Zdeb, Nadiia Rebrova, Radosław Lisiecki and Przemysław Jacek Dereń *

Institute of Low Temperature and Structure Research, Polish Academy of Science, Okólna Street 2, 50-422 Wrocław, Poland; p.zdeb@intibs.pl (P.Z.); n.rebrova@intibs.pl (N.R.); r.lisiecki@intibs.pl (R.L.)

* Correspondence: p.deren@intibs.pl

Table S1. Lattice parameters and unit cell volume of $KLaF_4:Pr^{3+}$ nanoparticles.

	a **(Å)**	***b*** **(Å)**	***c*** **(Å)**	***V*** **(Å³)**
$KLaF_4$	6.3299	3.8509	15.6801	382.21
$KLaF_4$:0.1%Pr^{3+}	6.3290	3.8509	15.6799	382.15
$KLaF_4$:0.5%Pr^{3+}	6.3285	3.8506	15.6755	381.98
$KLaF_4$:1%Pr^{3+}	6.3232	3.8501	15.6723	381.54
$KLaF_4$:1.5%Pr^{3+}	6.3228	3.8499	15.6699	381.44
$KLaF_4$:2%Pr^{3+}	6.3227	3.8498	15.6696	381.41

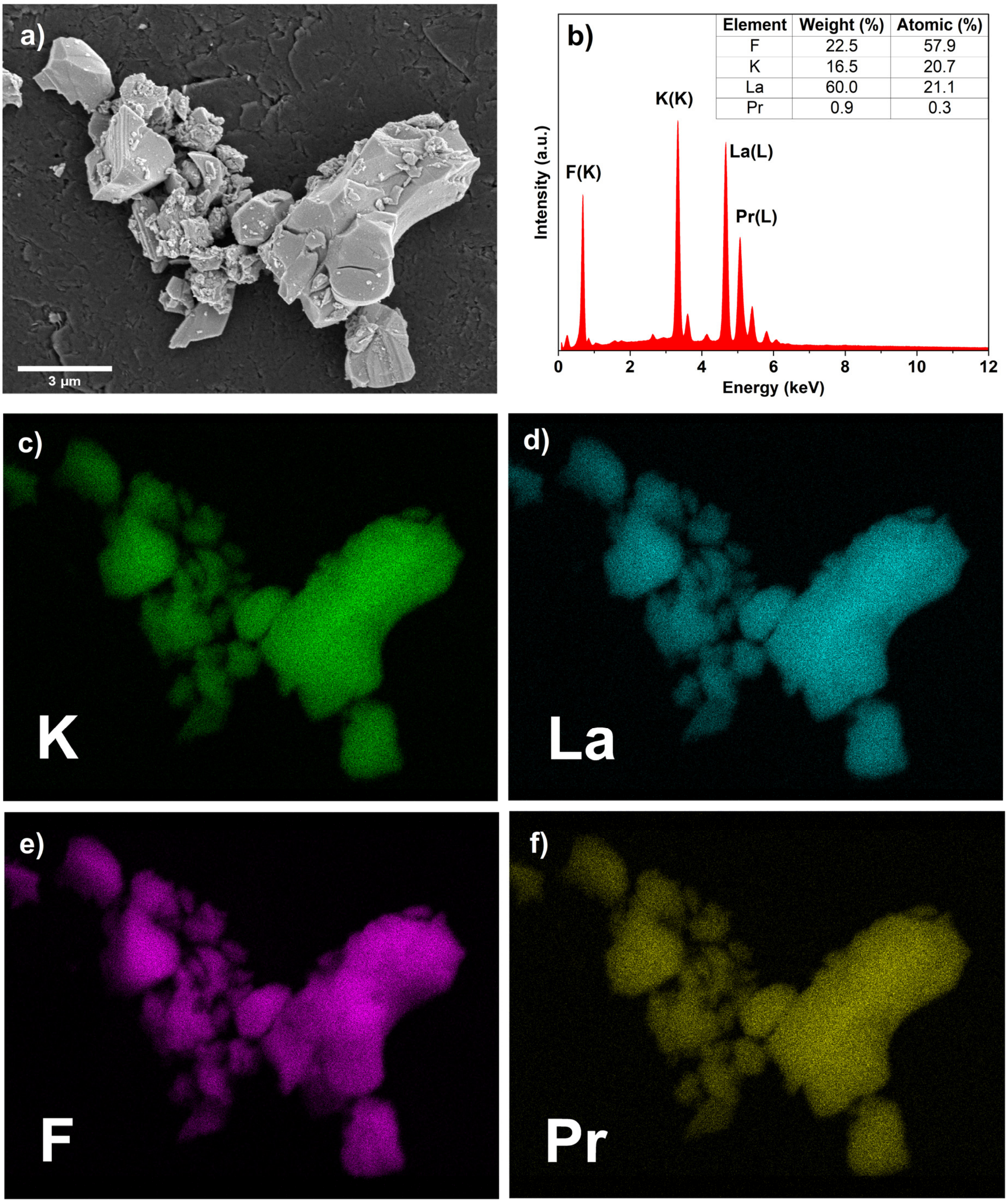

Figure S1. a) SEM image of analyzed grain. b) EDS spectrum of $KLaF_4$:1.5%Pr^{3+} sample; inset shows weight and atomic percentage of elements in the matrix. c-f) EDS element mapping.

Table S2. Information about bands observed in the excitation and emission spectra of $KLaF_4$:1%Pr^{3+} measured in the UV range.

**Excitation**				
**Transition**	***E* (cm^{-1})**	**λ (nm)**	**FWHM (cm^{-1})**	**Peak number**
	52880	189	3486	1
	55872	179	5291	2
$^3H_4 \rightarrow 4f^15d^1$	60480	165	2808	3
	62408	160	2177	4
	64472	155	4177	5
**Emission**				
**Transition**	***E* (cm^{-1})**	**λ (nm)**	**FWHM (cm^{-1})**	**β_{ex} [%]**
$^1S_0 \rightarrow {}^3H_4$	46189	216	1177	3.8
$^1S_0 \rightarrow {}^3H_6$	42463	236	1140	1.8
$^1S_0 \rightarrow {}^3F_3$	39761	252	845	17.8
$^1S_0 \rightarrow {}^1G_4$	36630	273	742	25.8
$^1S_0 \rightarrow {}^1D_2$	29630	338	615	10.9
$^1S_0 \rightarrow {}^1I_6$	24661	406	776	39.8
$^3P_0 \rightarrow {}^3H_4$	20640	484	315	

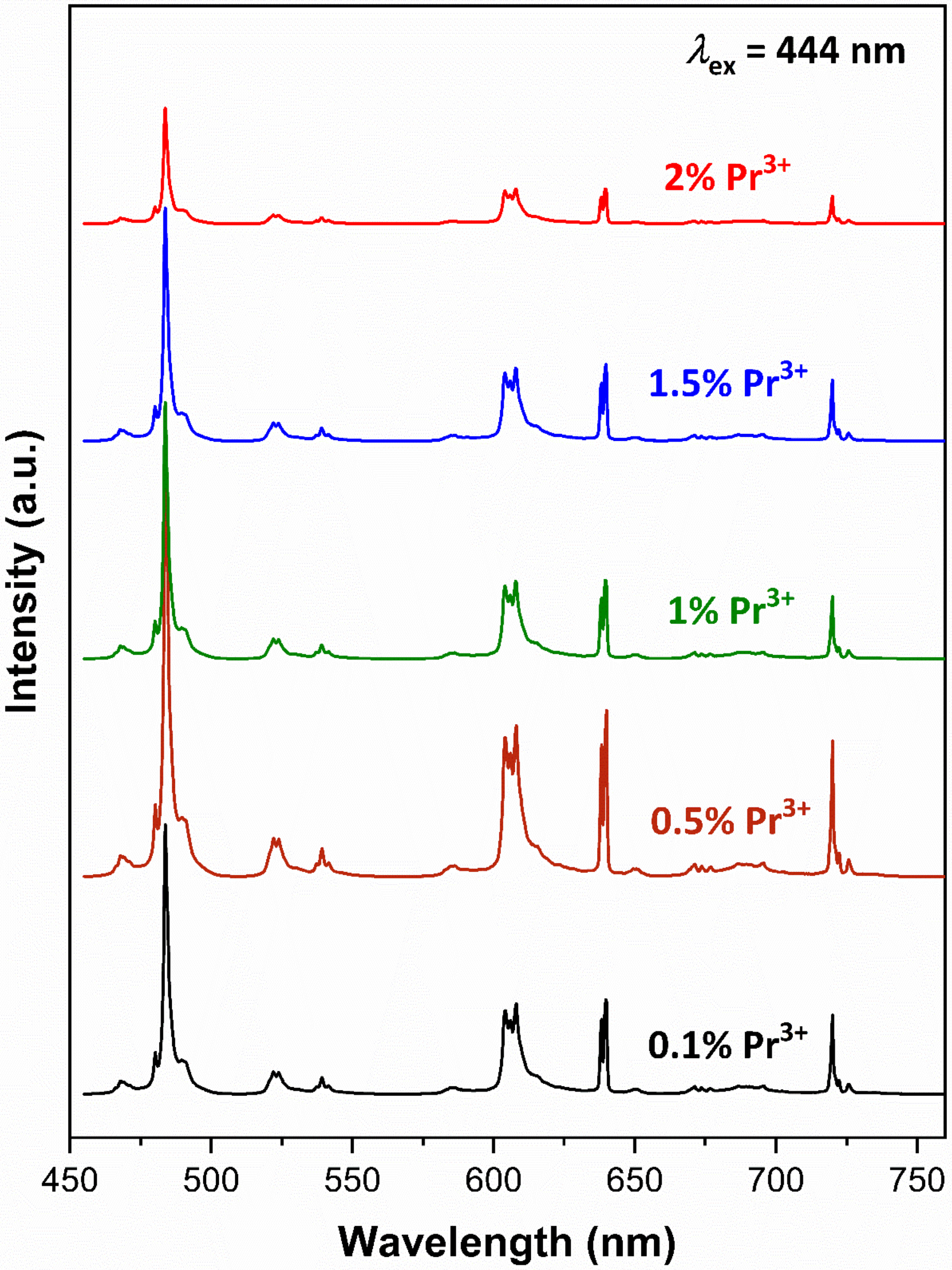

Figure S2. Emission spectra of $KLaF_4$ doped with different Pr^{3+} ion concentrations, measured under 444 nm excitation.

Table S3. Experimental energies of Pr^{3+} Stark levels in $KLaF_4$ host.

$^{2S+1}L_j$	Experimental Stark-levels energy (cm^{-1})	Experimental number of levels	Theoretical number of levels
$^3H_4$	0, 114, 147, 186, 222	5	9
$^3H_5$	2208, 2271, 2361	3	11
$^3H_6$	4275, 4321, 4374, 4425, 4457	5	13
$^3F_2$	5143, 5146, 5175, 5188	5	5
$^3F_3$	6410, 6432, 6454, 6509, 6570, 6581*	6	7
$^3F_4$	6915, 6921, 6923, 6943, 6969	5	9
$^1G_4$	9694*	1	9
$^1D_2$	16775*	1	5
$^3P_0$	20809	1	1
$^3P_1$	21352, 21411, 21431	3	3
$^3P_2$	22506, 22524, 22586, 22597	2	5
$^1S_0$	46387*	1	1

* Energies calculated based on the room temperature emission spectra (**Error! Reference source not found.**b and 5b).

Table S4. Comparison of the maximum relative temperature sensitivities ($S_{R\,MAX}$ at given T_{MAX}) of different Ln^{3+}-based luminescence thermometers working in the low-temperature range.

Host	Dopant	T_{MAX} [K]	$S_{R\,MAX}$ (%K^{-1})	Reference
$KLaF_4$	Pr^{3+}	140	1.70	This work
β-$NaYF_4$	Pr^{3+}	120	≈ 5	[1]
Y_2O_3	Nd^{3+}	123	1.51	[2]
$NaYF_4$	Nd^{3+}	203	16.3	[3]
fluoroindate glass	Er^{3+}	152	2.8	[4]
$NaGdF_4$	Yb^{3+}	125	≈ 1.2	[5]
La_2MgTiO_6	Cr^{3+}, V^{4+}	165	1.96	[6]
[GA]Mn(HCOO)3	Cr^{3+}	100	1.20	[7]
$(Me_2NH_2)_3[Eu_3(FDC)_4(NO_3)_4]\cdot 4H_2O$	Eu^{3+}	170	2.7	[8]

REFERENCES:

1. Zhou, S.; Jiang, G.; Wei, X.; Duan, C.; Chen, Y.; Yin, M. Pr^{3+}-Doped β-$NaYF_4$ for Temperature Sensing with Fluorescence Intensity Ratio Technique. *J. Nanosci. Nanotechnol.* **2014**, *14*, 3739–3742.
2. Kolesnikov, I. E.; Kalinichev, A. A.; Kurochkin, M. A.; Mamonova, D. V.; Kolesnikov, E. Y.; Lähderanta, E.; Mikhailov, M. D. Bifunctional heater-thermometer Nd^{3+}-doped nanoparticles with multiple temperature sensing parameters. *Nanotechnology* **2019**, *30*, 145501.
3. Trejgis, K.; Ledwa, K.; Bednarkiewicz, A.; Marciniak, L. A single-band ratiometric luminescent thermometer based on tetrafluorides operating entirely in the Infrared Region. *Nanoscale Adv.* **2022**, *4*, 437–446.
4. Haro-González, P.; León-Luis, S. F.; González-Pérez, S.; Martín, I. R. Analysis of Er^{3+} and Ho^{3+} codoped fluoroindate glasses as wide range temperature sensor. *Mater. Res. Bull.* **2011**, *46*, 1051–1054.
5. Zheng, S.; Chen, W.; Tan, D.; Zhou, J.; Guo, Q.; Jiang, W.; Xu, C.; Liu, X.; Qiu, J. Lanthanide-doped $NaGdF_4$ core-shell nanoparticles for non-contact self-referencing temperature sensors. *Nanoscale* **2014**, *6*, 5675–5679.
6. Stefańska, D.; Bondzior, B.; Vu, T. H.; Grodzicki, M.; Dereń, P. J. Temperature sensitivity modulation through changing the vanadium concentration in a La_2MgTiO_6:V^{5+},Cr^{3+} double perovskite optical thermometer. *Dalton Trans.* **2021**, *50*, 9851–9857.
7. Stefańska, D.; Kabański, A.; Vu, T. H.; Adaszyński, M.; Ptak, M. Structure, luminescence and temperature detection capability of $[C(NH_2)_3]M(HCOO)_3$ (M = Mg^{2+}, Mn^{2+}, Zn^{2+}) hybrid organic–inorganic formate perovskites containing Cr^{3+} ions. *Sensors* **2023**, *23*, 6259.
8. Li, L.; Zhu, Y.; Zhou, X.; Brites, C. D.; Ananias, D.; Lin, Z.; Paz, F. A.; Rocha, J.; Huang, W.; Carlos, L. D. Visible-light excited luminescent thermometer based on single lanthanide organic frameworks. *Adv. Funct. Mater.* **2016**, *26*, 8677–8684.
